# Using density of antecedent events and trajectory path analysis to investigate family-correlated patterns of onset of bipolar I disorder: a comparison of cohorts from Europe and USA

**DOI:** 10.1186/s40345-021-00234-4

**Published:** 2021-10-01

**Authors:** Jan Scott, Florence Vorspan, Josephine Loftus, Frank Bellivier, Bruno Etain

**Affiliations:** 1 Institute of Neuroscience, Newcastle University, Newcastle upon Tyne, UK; 2 Université de Paris, Paris, France; 3 AP-HP, Département de Psychiatrie et de Médecine Addictologique, GH Saint-Louis-Lariboisière-Fernand-Widal, DMU Neurosciences Tête et Cou, Paris, France; 4 Inserm UMRS 1144, Université de Paris, Paris, France; 5 Centre Expert Trouble Bipolaire, Hospital Princesse Grace, Monaco, Monaco

**Keywords:** Bipolar I disorder, Comorbidities, Trajectories, Antecedents, Cohorts, Family history

## Abstract

**Background:**

Major contributors to the global burden of bipolar disorders (BD) are the early age at onset (AAO) and the co-occurrence of non-mood disorders before and after the onset of BD. Using data from two independent cohorts from Europe and the USA, we investigated whether the trajectories of BD-I onset and patterns of psychiatric comorbidities differed in (a) individuals with or without a family history (FH) of BD, or (b) probands and parents who both had BD-I.

**Methods:**

First, we estimated cumulative probabilities and AAO of comorbid mental disorders in familial and non-familial cases of BD-I (Europe, n = 573), and sex-matched proband-parent pairs of BD-I cases (USA, n = 194). Then we used time to onset analyses to compare overall AAO of BD-I and AAO according to onset polarity. Next, we examined associations between AAO and polarity of onset of BD-I according to individual experiences of comorbidities. This included analysis of the density of antecedent events (defined as the number of antecedent comorbidities per year of exposure to mental illness per individual) and time trend analysis of trajectory paths plotted for the subgroups included in each cohort (using R^2^ goodness of fit analysis).

**Results:**

Earlier AAO of BD-I was found in FH versus non-FH cases (log rank test = 7.63; p = 0.006) and in probands versus parents with BD-I (log rank test = 15.31; p = 0.001). In the European cohort, AAO of BD-I was significantly associated with factors such as: FH of BD (hazard ratio [HR]: 0.60), earlier AAO of first non-mood disorder (HR: 0.93) and greater number of comorbidities (HR: 0.74). In the USA cohort, probands with BD-I had an earlier AAO for depressive and manic episodes and AAO was also associated with e.g., number of comorbidities (HR: 0.65) and year of birth (HR: 2.44). Trajectory path analysis indicated significant differences in density of antecedents between subgroups within each cohort. However, the time trend R^2^ analysis was significantly different for the European cohort only.

**Conclusions:**

Estimating density of antecedent events and comparing trajectory plots for different BD subgroups are informative adjuncts to established statistical approaches and may offer additional insights that enhance understanding of the evolution of BD-I.

**Supplementary Information:**

The online version contains supplementary material available at 10.1186/s40345-021-00234-4.

## Background

Globally, bipolar disorders (BD) are ranked the sixth most burdensome disease in working age adults (Gore et al. 2011). Key reasons for the substantial morbidity are that 75% of BD onsets occur at age < 30, and that BD is highly comorbid (Angst et al. 2002; Merikangas et al. 2007; Merikangas et al. 2008). These issues are amplified in individuals with a family history (FH) of BD who have an increased risk of developing BD, to experience an earlier age at onset (AAO) of BD, and to report high rates of mood and non-mood disorders during childhood and adolescence compared with control populations (e.g., Duffy et al. 2019; Hafeman et al. 2017; Lau et al. 2018). Emerging evidence confirms that many comorbidities occur before the first BD episode, i.e., they are antecedents of BD-onset not just complications of the disorder (e.g., Faedda et al. 2014; Hafeman et al. 2017; Carpenter et al. 2020).

For example, several meta-analyses report the lifetime rates of different psychiatric disorders in BD populations and some explore comorbidities in those at high risk of developing BD in the future (e.g., Lau et al. 2018; Pavlova et al. 2015; Messer et al. 2017). Whilst these confirm high rates of mood and non-mood disorders, the sequence of onset of the different comorbidities has only been examined consistently in prospective studies of offspring of parents with BD (e.g., Duffy et al. 2019; Mesman et al. 2013; Hafeman et al. 2017). The findings suggest that, for example, most anxiety disorders may occur earlier than mood problems and precede substance misuse (Lau et al. 2018).

However, many well-known offspring and youth cohort studies are still ongoing, and so large proportions of the research participants have not yet been followed through the entire peak age range for onset of BD (and/or for non-mood comorbidities) (e.g. Duffy et al. 2019; Lee et al. 2018; Koenders et al. 2020). Additionally, even though the study samples may be relatively large, the total number of BD cases is small in the offspring or controls (e.g. Duffy et al. 2019; Koenders et al. 2020). The potential consequence of this scenario is that these studies cannot yet fully account for the pattern of associations between comorbidities and being diagnosed with BD, nor any differences in comorbidity rates and the timing of onset of BD in familial and non-familial cases.

Studies of offspring and other cohorts have been undertaken over a number of decades, and have generated a number of other interesting observations. For example, when current and past research are examined side by side it appears that the AAO of BD may be decreasing (e.g., Golmard et al. 2016; Scott et al. 2018). Several explanations have been proposed for this phenomena. These include the possibility that a lower AAO of BD could be linked to increasing rates of psychiatric comorbidities and/or that it might be linked to higher familial loading and/or childhood adversity (e.g., Post et al. 2015). However, no study to date has determined how the pattern of onsets of comorbidities may be linked with AAO of BD in cases with or without a family history of BD. Likewise, very few studies have examined similarities in the evolution of BD in probands and parents, so it is not clear if there is concordance in the pattern of comorbidities across generations (Dierker et al. 1999).

Overall, our examination of previous research highlighted that gaps exist in the understanding of cumulative rates of and AAO of comorbidities observed in individuals who progress to a diagnosis of BD. Also, there are uncertainties regarding associations between the trajectories of comorbidities experienced (i.e., sequence and timing of onset of non-mood disorders) and their relationship to AAO of BD in subpopulations with different levels of premorbid risk (e.g. familial versus non-familial BD) (Carpenter et al. 2020). Further, we noted that many existing studies of comorbidity only focus on specific diagnoses or classes of diagnoses (such as anxiety disorders) without exploring whether other markers of illness burden (e.g., number and timing of onset of all the comorbidities experienced) are associated with polarity of onset or AAO of BD (Merikangas et al. 2017).

We decided to undertake a project to address some of these gaps but were mindful that there are several difficulties in undertaking research on trajectories of comorbidities in individuals with familial versus non-familial BD and/or between generations within the same family. The three most common problems identified in previous research were: the reliability of BD diagnoses; the quality of assessments of family history (FH); and the rigour of reporting of individual longitudinal psychiatric histories (Leckman et al. 1982; Wacholder et al. 1991; Duffy et al. 2011). To address these concerns, we used data from existing cohort studies that employed reliable and valid assessment tools and that were sufficiently similar methodologically to allow some cross-study conclusions to be drawn. We applied for access to two assembled cohorts of BD cases from which we extracted data on individuals who met recognized, international diagnostic criteria for BD-I (we chose to focus on this subtype, as the diagnosis of BD-I is the third most reliable diagnosis in psychiatry) (First 2016). Further, the cohort members: (i) were recruited to studies that paid specific attention to familiality, namely genetics studies in Europe (cases with/without a FH of BD) and the USA (proband-parent pairs of BD-I cases: PRB-PAR), (ii) were beyond the peak age range for risk of onset of BD-I (to allow us to carefully assess comorbidities that occur as antecedents versus consequences of BD-I onset), and (iii) the evaluations of best-estimate diagnoses and AAO of lifetime DSM IV comorbidities were undertaken using the same, well-established structured clinical interview assessment undertaken by trained researchers.

In this article, we investigate questions related to three themes-

(i) Patterns (type and AAO) of comorbid conditions in familial cases of BD-I and within-family correlation:
  Do familial cases of BD-I (FH) demonstrate any differences in the type or AAO of lifetime comorbid mental disorders compared with non-familial cases (No FH; sometimes referred to as phenocopies or as ‘sporadic ‘BD cases)?
  and
  Do probands with BD-I demonstrate any differences in the type or AAO of lifetime comorbid mental disorders compared with their same-sex biological parent with BD (PRB-PAR pairs)?
(ii) Comorbid conditions and AAO of BD-I and AAO according to onset polarity:
  In the two study cohorts (FH/No FH; PRB-PAR), is there any association between exposure to any comorbid mental disorder(s) and the AAO of BD-I and/or polarity of first episode of BD-I?
  and
  In each cohort, what are the similarities and differences in the overall pattern of comorbidities between subgroups?
(iii) Density of antecedent events and trajectory plots:
  An additional goal of this project was to explore new ways of presenting comparative data that would be easy to interpret or understand (e.g., for patients and families, those outside academic settings). So, we explored—Is it possible to represent the pattern of comorbidities using novel metrics such as density of antecedent events (number of antecedent comorbidities per year of exposure to mental illness per individual) and plots of trajectory paths?
  Do the estimates for antecedents and trajectories differ between subgroups included within each cohort?

## Method

The project was approved by the French medical ethics committee (Comité de Protection des Personnes- IDRCB_AO1465_50_VI-Pitié Salpêtrière) and follows the Strengthening the Reporting of Observational Studies in Epidemiology guidelines (STROBE). Here, we summarize key elements of the methodology only, but Additional file 1: Appendix 1 includes further details of the protocol (including rationale for selection of datasets, statistical procedures, etc.) and separate STROBE checklists for each study cohort.

### Sampling and data extraction

#### Assembled cohorts

Written informed consent was obtained from all participants and de-identified data on demographics and clinical phenotypes were extracted from a European genetics database study (data from France) and a genetic linkage study database provided by the NIMH Repository and Genomics Resource in the USA (permissions to use data from NRGR Bipolar Disorder distribution 12.0 was granted to Scott and colleagues; access ID number: 5c9874082337f). Additional file 1: Appendix 1 highlights similarities and differences in data collection and recording between Europe and the USA (Potash et al. 2007; Etain et al. 2012).

Familial and Non-Familial Cases: The European dataset comprised information about individuals meeting DSM-IV criteria for BD-I who were recruited via three French university-affiliated psychiatry departments between 1994 and 2008. Individuals aged > 18 were interviewed using the French version of the Diagnostic Interview for Genetic Studies (DIGS) and Family Interview for Genetic Studies (FIGS) (Maxwell 1992; Nurnberger et al. 1994).

Sex-Matched Proband-Parent Pairs (PRB-PAR): From datasets that included individuals with BD, we identified adults aged > 18 at baseline assessment who were recruited to USA-based genetics linkage studies between 1991 and 2003 and assessed using the DIGS. We extracted data on pairs of cases, comprising probands with a DSM-IV diagnosis of BD-I matched to their same sex biological parent with DSM-IV diagnosis of BD-I.

#### Illness trajectories

Clinical data collected using the DIGS allowed construction of individual patterns of exposure to comorbidities and illness trajectories. Information on comorbid disorders that fulfilled DSM-IV diagnostic criteria and the AAO of each full-threshold disorder experienced was recorded. We classified polarity of onset of BD-I as depressive (first mood episode met DSM-IV criteria for a major depressive episode) or manic (first mood episode met DSM-IV criteria for a manic and/or mixed episode). As in similar research, AAO were estimated from DIGS, case note and informant information (Egeland et al. 1987). Combining data from individual profiles allowed plots of illness trajectories for each subgroup to be generated (Thomas et al. 2009).

We further examined patterns of comorbidities by estimating the total number of comorbid conditions reported per individual (i.e., the combination of pre- and post-onset of BD), and the number of comorbid conditions that occurred prior to the onset of onset of BD-I per individual (Vaidyanathan et al. 2011). We then estimated the density of antecedent events, which represents the number of events per year of exposure to mental disorders prior to BD-I onset for each individual (where illness exposure is calculated as the time interval between AAO of the first DSM-IV diagnosis and AAO of the first episode of BD-I; and only comorbidities preceding BD onset are considered). As such, this variable encompasses several key elements regarding the nature, pattern and timing of comorbidities and offers a valuable proxy that we employed in our exploration of trajectory paths.

### Statistical analysis

Analyses were undertaken using RStudio version 3.5.3, and SAS version 9.4. A priori we defined statistical significance as a p value of 0.05 or less.

#### Descriptive analysis

Categorical data regarding cumulative probabilities of DSM IV disorders were described using counts and percentages; continuous data regarding AAO of each diagnosis were described using medians with inter-quartile ranges (IQR). Basic characteristics were compared between subgroups using non-parametric univariate analyses (analyses for related samples were used for PRB-PAR).

#### Time to event (onset of BD-I) analysis

Kaplan Meier estimators tested subgroup differences in AAO of BD-I with statistical significance established using Mantel Haenszel log-rank tests. For the European cohort, we used an accelerated failure time (AFT) model to test effects of key covariates on the time to onset of BD-I (Wei 1992). An AFT model assumes that the effect of covariates (such as AAO of first comorbidity) may act multiplicatively with respect to the AAO of BD-I. The hazard ratio (HR) estimates, in effect, represent a time ratio with 95% confidence intervals (CI). As recommended, we employed generalized estimating equations (GEE) for analyses in PRB-PAR (Zeger et al. 1988). All these analyses included sex and year of birth as covariates.

#### Exploratory trajectory analysis

We used a time trend design to estimate longitudinal changes in health/illness status, with age representing the underlying time scale (Bailey et al. 2005; Thomas et al. 2009). Trend estimation was used to relate observations of interest (DSM IV diagnoses) to the time at which they occur (AAO). We first produced bubble plots to demonstrate the sequence and rates of comorbidities over time (using GraphPad Prism 8). The size of each bubble represents the cumulative probability of a DSM IV disorder occurring within a subgroup. The order in which the bubbles occur on the trajectory path represents the sequencing of onsets of each reported comorbidity. The location of the bubble on the y-axis identifies the median AAO of that disorder within the subgroup (the location of the bubbles on the x-axis approximates to the timing of onset of a disorder within the total course of illness) (Loftus et al. 2020).

Using the bubble plots, we then plotted a logistic trendline; this best-fitted curve line is recommended as the most appropriate option for analogous longitudinal data (Ho-Trieu and Tucker 1990; Thomas et al. 2009). The goodness of fit of the trajectory was estimated using the least square fitting process, where R^2^ represents the fraction of the variance explained by the fitted trendline (R^2^ range = 0–1; a value of one would indicate a perfect fit of data to the path of the trajectory curve) (Zheng 2000; Weisstein 2020). The R^2^ for subgroups were compared statistically using a Z transformation (see Additional file 1: Appendix 1 for details). To further illuminate patterns represented in the bubble plots for each subgroup within each cohort, we analyzed differences in the number of antecedent comorbidities (without consideration of time frame) and in the density of antecedent events.

## Results

For each cohort we show one table and one figure in the main text, with two additional tables and figures in the supplementary materials (Additional files 2, 3: Appendices 2 and 3). As the paper is primarily focused on trajectory paths and R^2^ analyses, we only briefly report AFT and GEE findings.

### Familial (FH) versus non-familial (no FH) BD-I cases

As shown in Table 1, the European cohort comprised 573 individuals; 322 were female (56.2%) and 209 (36.5%) had a confirmed FH of BD. The sample median AAO of BD-I was 22.0. Over half the cohort (N = 301; 52.5%) reported ≥ 1 comorbid DSM IV diagnosis (irrespective of mood disorder episodes) with a median AAO of the first full-threshold disorder of 18.0. The commonest non-mood comorbid condition was alcohol abuse (N = 124; 21.7%) and the rarest was OCD (N = 28; 5%). Social phobia had the youngest median AAO (15.0 years), whilst alcohol dependence had the oldest median AAO (28.5 years).

**Table 1:** Characteristics of 573 individuals with BD-I (209 with a family history and 364 without a family history of BD)

	Cumulative probability		Median age at onset in years (interquartile range)	
	Number	(%)^c^		
Females	322	56.2		
Family history of BD	209	36.5		
Age at onset of BD			22.0 (18.0, 30.5)	
Duration of BD			16.0 (11.5, 25.3)	
Lifetime comorbidities:				
≥ 1 comorbid mental disorder^a^	301	52.5		
Age at onset of first mental disorder^b^			18.0 (14.3, 25.5)	
Social phobia	81	14.2	15.0 (11.5, 20.3)	
Specific phobia	49	8.5	15.5 (10.3, 22.5)	
Obsessive compulsive disorder	28	5.0	18.3 (14.0, 23.0)	
Generalized anxiety disorder	45	7.9	20.8 (17.0, 27.5)	
Agoraphobia	53	9.4	20.0 (15.3, 36.0)	
Panic disorder	108	18.9	22.0 (17.5, 32.3)	
Eating disorder	56	9.8	16.8 (14.0, 18.3)	
Cannabis abuse	88	15.4	17.8 (17.0, 20.5)	
Cannabis dependence	38	6.7	18.3 (16.5, 21.8)	
Alcohol abuse	124	21.7	23.5 (18.3, 35.0)	
Alcohol dependence	45	7.8	28.5 (21.3, 35.0)	

^a^Comorbid mental disorder refers to any DSM IV diagnosed disorder that occurred (irrespective of BD-I mood episodes);

^b^First mental disorder refers to a clinical condition that met criteria for a full-threshold DSM IV diagnosis

^c^Denominator for comorbid disorders ranges from n = 549 to n = 573 (due to small number of sporadic missing values)

Comparison between subgroups demonstrated that about 61% of individuals with a FH and 57% without a FH (i.e., No FH group) reported a depressive polarity of onset of BD-I (Additional file 2: Table S1, Appendix 2). The median AAO was a statistically significant different for depressive onset polarity (FH: 20.0; No FH: 22.8; p < 0.007), but not for manic polarity of onset (FH: 22.0; No FH: 23.5). As shown in Additional file 2: Table S1, there were few differences in rates of specific comorbidities except for cannabis dependence (FH: 9.2%; No FH: 3.8%; p < 0.008), and few differences in median AAO of comorbidities, except for agoraphobia (FH: 20.0; No FH: 26.5; p < 0.046). However, Additional file 2: Table S2 shows that the FH group reported significantly more comorbidities in total (i.e., pre-and-post-onset of BD-I) and significantly more occurring prior to BD-I onset.

Time to event analysis showed that familial BD was associated with a significantly earlier AAO of BD-I (log rank test = 7.63; p = 0.006). Analysis of AAO of BD-I according to polarity demonstrated that for manic onset, the best predictors of earlier AAO of mania was the presence of a FH of BD (HR: 0.60; 95% CI: 0.38, 0.94) and earlier AAO of first DSM IV diagnosis (HR: 0.97; 95% CI: 0.95, 0.99). For depressive polarity of onset, the best predictors of earlier AAO of BD-I were earlier AAO of first DSM IV diagnosis (HR: 0.93; 95% CI: 0.89, 0.98) and greater total number of comorbidities (HR: 0.74; 95% CI: 0.56, 0.95).

**Fig. 1:**
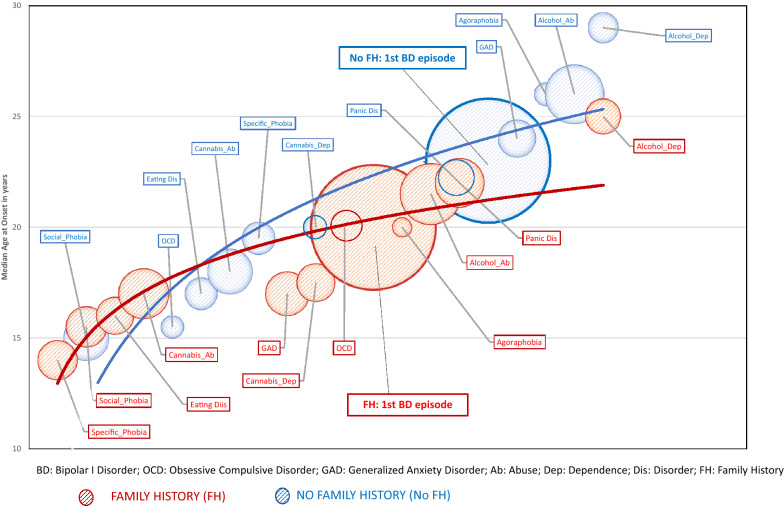
Trajectories of evolution of BD-I in groups with (FH) or without (no FH) a family history of Bipolar I Disorder (BD). In the bubble plot, the size of the bubble represents the proportion of the group who experience a particular disorder, the position of the bubble vertically gives the median AAO, whilst the position on the horizontal axis approximates to the timing of onset of a disorder in the interval between the onset of the first full threshold mental disorder and the onset of the last comorbidity. For example: in the FH subgroup, the median AAO of the first mental disorder (specific phobia) is about 13–14 years, whilst the median AAO of BD-I is about 20 years. The curve fits less well to the trajectory of comorbidities for the period post-onset of BD-I in the FH group. In the No FH group, the median AAO of the first mental disorder (social phobia) is about 14–15, whilst the median AAO of BD-I is about 22 years. In the No FH group, the curve fits less well to the trajectory path of those antecedent comorbidities with the earliest AAO. The bubbles for BD-I represent 100% in both subgroups. The location of that bubble for FH is lower on the vertical axis, indicating the disorder has an earlier AAO than in the ‘No FH’ group. Also, as can be observed, there appears to be a more obvious clustering of (relatively) early AAO antecedent comorbid disorders within a briefer time interval in the FH group, compared with the No FH group. There are a smaller number of between-group differences in the sequence and onset of different mental disorders

Figure 1 shows the bubble plots and best-fitted trajectory curves for the FH and No FH subgroups (separate plots that show depressive and manic polarities and mathematical formulae describing the trajectories are shown in Additional file 3: Fig. S1 in Appendix 3). As can be seen in Fig. 1, the trajectory curve for the FH subgroup looks flatter and the density of antecedents appears greater (and comorbidities cluster at an earlier age). These observations were confirmed by statistical analyses. The R^2^ for the trajectory curves for the two subgroups were significantly different (FH: 0.88, 95% CI: 0.85, 0.91; No FH: 0.94, 95% CI: 0.92, 0.95; Z = − 2.98; p = 0.01). As shown in Additional file 2: Table S2, individuals with a FH reported significantly more antecedents compared with those with No FH (grouped medians: FH: 1.16; No FH: 0.47; p < 0.04) and a significantly higher density of antecedents per individual (grouped medians: FH: 0.078; No FH: 0.051; p < 0.018).

### Proband-parent pairs

As shown in Table 2, the USA cohort comprised 184 individuals (92 PRB-PAR pairs); 100 individuals were female (54.3%). The median AAO of BD-I was 19.3 and median duration of BD-I at the time of interview assessment was 20.3 years. Over half the cohort (N = 95; 51.6%) reported ≥ 1 comorbid DSM IV mental disorder (irrespective of mood disorder episodes) with a median AAO of the first full-threshold disorder of 15.0 years. The commonest comorbid condition was AUD (N = 58; 31.5%) and the rarest was OCD (N = 6; 3.3%). Specific phobia had the earliest median AAO (9.0 years), whilst AUD had the latest median AAO (28.3 years).

**Table 2:** Characteristics of 184 individuals with BD-I (92 sex-matched proband-parent pairs)

	Cumulative probability		Median age at onset in years (interquartile range)	
	Number	(%)^c^		
Females	100	54.3		
Age at onset of BD			19 (15.3, 26.0)	
Duration of BD			20.3 (10.3, 35.8)	
Lifetime comorbidities:				
≥ 1 comorbid mental disorder^a^	95	51.6		
Age at onset of first mental disorder^b^			15.0 (10.5, 17.8)	
Social phobia	9	5	14.5 (9.0, 20.3)	
Specific phobia	14	7.5	9.0 (6.3, 13.8)	
Obsessive compulsive disorder	6	3.3	17.5 (11.5, 28.0)	
Generalized anxiety disorder	30	16.3	21.8 (17.0, 27.3)	
Agoraphobia	12	6.5	17.8 (12.3, 28.0)	
Panic disorder	33	18.0	23.0 (15.5, 32.3)	
Eating disorder	11	6.0	17 (12.8, 22.5)	
Substance use disorder	23	12.5	18.0 (15.8, 25.3)	
Alcohol use disorder	58	31.5	28.3 (23.0, 37.5)	

^a^Comorbid mental disorder refers to any DSM IV diagnosed disorder that occurred (irrespective of BD-I mood episodes);

^b^First mental disorder refers to a clinical condition that met criteria for a full-threshold DSM IV diagnosis

^c^Denominator for comorbid disorders ranges from n = 175 to n = 184 (due to small number of sporadic missing values)

Comparison between subgroups (Additional file 2: Table S3 in Appendix 2) demonstrated that 61% of both probands and parents reported depressive polarity of onset of BD-I. There were statistically significant differences in the median AAO of BD-I between PRB-PAR pairs for both depressive onset polarity (PRB: 17.3PAR: 23.0; p < 0.001) and manic onset polarity (PRB: 20.0; PAR: 24.3; p < 0.001). There were few differences regarding specific comorbidities, except for trends for OCD to be more common in PRB (PRB: 5.4%; PAR 1.1%; p = 0.067) and the median AAO of AUD to be younger in PRB (PRB: 26.0; PR: 33.0; p = 0.052).

Time to event analysis confirmed that PRB had a significantly earlier AAO of BD-I than PAR (log rank test = 15.31; p = 0.001). Median AAO of BD-I in those with a manic onset was associated with number of comorbidities (HR: 0.65; 95% CI 0.44, 0.95) and year of birth (HR: 2.44; 95% CI: 1.58, 3.76), with later AAO of BD-I was associated with earlier year of birth. Median AAO in those with a depressive onset polarity was associated with the same two variables (HR for comorbidities: 0.44; 95% CI: 0.29, 0.68; HR for year of birth: 1.29; 95% CI: 1.04, 1.58); sex was of borderline significance in this model (HR for male sex: 1.28; 95% CI: 0.94, 1.68). It was noted that PRB/PAR status was not a significant covariate in either model.

**Fig. 2:**
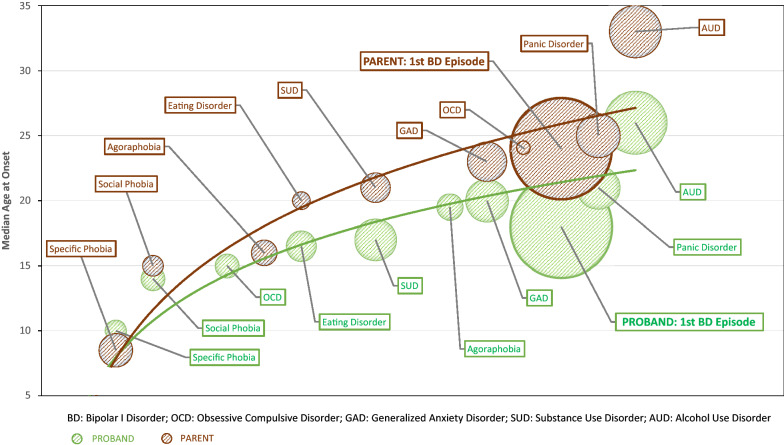
Trajectories of evolution of BD-I in probands and parents. In the bubble plot, the size of the bubble represents the proportion of the group who experience a particular disorder, the position of the bubble vertically gives the median AAO, whilst the position on the horizontal axis approximates to the timing of onset of a disorder in the interval between the onset of the first full threshold mental disorder and the onset of the last comorbidity. It is notable that the median AAO of the first mental disorder (specific phobia) is similar in the Proband and Parent subgroups, and that the sequence of onset of disorders (except, e.g. OCD) is similar. The size of the bubbles is somewhat similar, but AAOs of comorbidities begin to occur earlier in the Proband subgroup, and median AAO of BD-I is clearly earlier than in the Parent subgroup. Both curves show the fit for the trajectory curve is less for comorbidities that occur post-onset of BD-I

Figure 2 shows the bubble plots and best-fitted trajectory curves for the PRB and PAR subgroups (also see Additional file 3: Fig. S2 in Appendix 3). The path of the trajectory curves demonstrates similar AAO of the first mental disorder, but an earlier median AAO for BD-I for PRB compared with PAR. However, the R^2^ for the trajectory curves for each subgroup were not significantly different (PRB: 0.90, 95% CI: 0.85, 0.93; PAR: 0.92, 95% CI: 0.88, 0.95; Z = 0.97; p = 0.33). As shown in Additional file 2: Table S4, PRB and PAR did not differ significantly in number of antecedents (grouped medians- PRB: 1.21; PAR: 0.96; p = 0.13). However, as might be expected given the earlier AAO of BD, PRB had a significantly higher density of antecedents per individual (grouped medians- PRB: 0.088; PAR: 0.071; p < 0.04).

## Discussion

This study used a series of planned analyses to explore the relationships between psychiatric comorbidities occurring before and after the onset of BD-I in groups with different premorbid levels of risk. We found that few between-group differences were identified by basic summary estimates of cumulative probabilities or AAO of specific comorbidities (except e.g. for the prevalence of cannabis dependence in FH cases and the AAO of AUD in PRB cases). We formed the opinion that univariate analyses cannot tell the complete story of the inter-relationship between comorbidities in BD. It was clear that more intriguing findings emerged when analyses are extended to consider the overall burden of comorbidities and whether the total exposure and/or timing of exposures to comorbidities is associated with AAO of BD. For example, we found that most FH and PRB cases experience ≥ 1 non-mood disorder prior to the onset of BD, and that the increased illness burden associated with all comorbidities appears to be due to incremental differences in the cumulative probability and/or AAO in these two subgroups compared with the other study groups. Furthermore, in the European cohort, AAO of the first DSM IV diagnosis was significantly associated with AAO of BD-I (irrespective of polarity), but only manic onset polarity was significantly associated with FH status. In contrast, in the USA cohort of PRB-PAR, AAO of BD-I was significantly associated with year of birth and burden of comorbidities irrespective of polarity of onset (and these covariates were more important than PRB-PAR status or AAO of first non-mood comorbidity). All of these findings were incorporated into the trajectory path analyses, which demonstrated that the density of antecedents was significantly higher in FH and PRB groups versus their comparators. However, the time trend R^2^ analysis was significantly different for the subgroups in the European cohort only. The latter finding may indicate that there are more dissimilarities in the evolution of BD between FH and No FH subgroups than between PRB-PAR pairs.

Although the current design differs from prospective offspring and/or youth cohort studies (which include BD and non-BD cases), some of the novel elements of our methodology make it possible to expand on ideas reported in that previous work (e.g., Angst et al. 2002; Mesmen et al. 2013; Etain et al. 2017; Merikangas et al. 2017; Scott et al. 2018; Duffy et al. 2019; Carpenter et al. 2020). For example, previous findings have demonstrated that AAO of BD-I in those with a FH is significantly earlier than those without a FH, but the addition of trajectory curve plots allows us to show that it is the pattern in the FH group that deviates more from the projected or hypothetical path (the R^2^ value indicates an inferior fit, or less predictable time trend curve for the FH compared with the No FH group). Likewise, in the PRB-PAR pairs, we showed that AAO of a first DSM IV disorder, sequence of comorbidities, number of antecedents are remarkably similar between offspring and parents. Whilst previous research has alluded to some concordance in comorbidities in PRB-PAR (Merikangas et al. 2017); to our knowledge, the current study is the first to demonstrate the similarities in these patterns and sequences using trajectory plots. As shown in Fig. 2, whilst the prevalence and AAO (size and location of bubbles), and trajectories (and R^2^) are comparable in many respects, the plots highlight the significant decrease in AAO of BD-I in PRB compared to PAR. These observations about the nature of divergences between PRB and PAR are confirmed by the estimated magnitude of the density of antecedent events per individual (which is higher in PRB). Unfortunately, we cannot further explore the similarities in patterns of illness or significant differences in AAO of BD-I in PRB-PAR using the current dataset. However, other researchers have suggested that the potential role of exposure to childhood adversity, higher genetic load, genetic anticipation, or birth cohort effects are worthy of further examination (Post et al. 2015; Golmard et al. 2016; Post et al. 2016; Etain et al. 2017; Scott et al. 2018; Duffy et al. 2019).

We propose that the estimating the ‘density of antecedent events’ can offer useful insights into individual (time-dependent) differences in illness exposure, giving a degree of personalization to the trajectories reported. Of course, it might be argued that it is a derived variable employed as a proxy for individual experiences of comorbidities and primarily reflects rather than adds to the data represented within the trajectory plots. However, we suggest that the ‘density of antecedent events’ captures the important notion of ‘counting people, not just disorders’ and is advantageous for several reasons of which we highlight just two examples (Vaidyanathan et al. 2011; Merikangas et al. 2017). First, data from epidemiological studies indicates that, in a given population, the sequence of disorders occurring across childhood, adolescence and early adulthood has some predictability (i.e. phobia/anxiety problems are more common in childhood; eating disorders in adolescence; alcohol misuse occurs slightly later). However, the prevalence rates or AAO data do not necessarily clarify whether a large number of individuals experience one disorder during childhood or adolescence, etc., or whether a small group of individuals experience several disorders, with onsets beginning in childhood and continuing throughout adolescence, etc. (Kim-Cohen et al. 2003; Copeland et al. 2011). Developing knowledge about the different patterns of comorbidity and illness trajectories requires new approaches to reporting the core data. For example, in the current study we found that rates of comorbidities may vary between the different subgroups examined, but we also note that, even within the FH and PRB groups, there were some BD cases who did not report any non-mood comorbidities. So, we think that finding ways to express individualized estimates as well as group or sample estimates will help research into any links between AAO of severe mental disorders in subgroups that that experience several disorders over time (so-called multi-morbidity) compared with those that have limited exposure to comorbidities (Angst et al. 2002; Merikangas et al. 2017). Second, by estimating individual experiences of antecedents separately, analyses can raise awareness of different patterns and timing of onset of comorbidity. Investigators can then consider whether individuals who only experience post-BD onset comorbidities have different characteristics from other individuals with the same BD subtype. In the current study, using such estimates of comorbidities enabled us to demonstrate that FH cases and PRB cases had a higher density of antecedents per individual, which we propose is compatible with notions of lower resilience (with increased vulnerability to multi-morbidity) and earlier AAO of BD in some individuals, as suggested by other researchers in the field (Hafeman et al. 2017; Duffy et al. 2019).

Another aim of this study was to find clinically informative methods for describing the trajectories of comorbidities and onset of BD-I, and to explore alternative ways to offer visual representations of findings. We did consider approaches used by other investigators (e.g., multistate models) and other figurative representations of pathways to BD, such as Sanking plots or schematic representations (that do not incorporate subgroup data) (e.g., Caspi et al. 2020). We do not consider that our current approach is superior, but we think time trend and R^2^ analyses have some merit. For example, this proved to be a pragmatic strategy for exploring complex data and the goodness of fit of trajectory models and graphically presenting the findings. Having made this observation, we accept that this exploratory approach is probably best be viewed as an adjunct rather than an alternative to established statistical analyses (such as time to event analyses, accelerated failure time model, generalized estimating equation, etc.).

We acknowledge several study limitations. Whilst the strict eligibility criteria for the original recruitment to the cohorts enhanced the reliability of data recordings, those criteria may reduce the clinical representativeness of our cases and the generalizability of findings. The data refer to assessments of retrospective events and, even though we applied the accepted, high-quality methodological strategies recommended for such studies, we know that recall bias may particularly affect AAO reporting and this could impact e.g., the validity of information regarding PRB and PAR. Also, there is increasing interest in subthreshold conditions, but these were not reported in the datasets obtained. However, the most important limitation is the relative lack of information regarding behavioural disorders. When the study cohorts were recruited, there was less awareness of the importance of associations between conditions such as ADHD and BD. (As noted in Additional file 1: Appendix 1, some of these conditions were assessed post-hoc, but the assessments employed meant we could not identify specific DSM IV diagnoses or AAO). Recent versions of the DIGS provide more detailed assessments of these conditions, but newer genetic datasets (such as those at NRGR) do not provide the detailed longitudinal information required to construct individual illness trajectories and/or had insufficient sample sizes. However, despite this study limitation, the AAO of first reported DSM IV disorders in the subgroups we studied are comparable to several other studies (Kim-Cohen et al. 2003; Golmard et al. 2016; Post et al. 2016; Etain et al. 2017; Lee et al. 2018; Caspi et al. 2020). Another obvious issue is that neither dataset included details about physical disorders (especially lacking information about AAO of any non-psychiatric problems). Given the known associations between physical disorders (such as cardio-respiratory and endocrine disorders) and BD, it would be of great interest to be able to incorporate medical conditions into the trajectory plots and density analyses to examine whether these also show any trends according to familial status or study location. Of course, these weaknesses highlight that replication and extension of the findings is required using independent datasets (Post et al. 2015; Golmard et al. 2016; Scott et al. 2018).

## Conclusions

This study of illness trajectories suggests that these plots can, for example, be employed to indicate the real-time clustering of comorbidities more precisely in individuals with a confirmed diagnosis of BD-I who showed differing levels of premorbid vulnerability to developing this disorder. Further, the plots may indicate which comorbidities are more likely to be antecedents of BD and which occur post-onset of BD, etc. The shape of the curves allows observation of the relative differences in AAO of a first DSM IV disorder, the clustering of comorbidities within specific age ranges, the likely sequence of onsets of comorbidities, and any links between these phenomena and AAO of BD. We believe these plots summarize large amounts of data in a user-friendly manner and may stimulate discussions with lay groups and stakeholders about the evolution of BD. The use of metrics such as density of antecedent events, time trend analysis and trajectory plots alongside other recognized statistical models may also generate ideas regarding new avenues for research on trajectories of BD.

Note to readers: We encourage readers to also review the extensive appendices provided with this paper that include details of sample selection, methodology and other basic findings that could not be included within the main text.

## Supplementary Information

**Additional file 1: Appendix 1**: STROBE checklists and additional details of methodology.

**Additional file 2: Appendix 2: Table S1.** Comparison of rates and age at onset for each comorbidity and polarity of onset of BD-I in individuals with a family history of BD (n=209) or without a family history of BD (n=364).** Table S2.** Comparison of patterns of comorbidities in individuals with a family history or without a family history of BD.** Table S3**. Comparison of rates and age at onset for each comorbidity and polarity of onset of BD-I in probands (n=92) and their sex-matched parents (n=92).** Table S4**. Comparison of patterns of comorbidities in probands and parents.

**Additional file 3: Appendix 3**:** Figure S1**. Trajectory paths for FH and non-FH.** Figure S2**. Trajectory plots for Probands and Parents.

## Data Availability

The authors confirm that the summary data for all variables supporting the findings of this study are included within the article and its supplementary materials. The raw data are being used at the lead research centres and form part of an ongoing programme of research and data are only made available upon written application to the relevant research committee.

## Abbreviations

AAO: Age at onset
ADHD: Attention deficit hyperactivity disorder
AFT: Accelerated failure time
AUD: Alcohol use disorder
BD: Bipolar disorders
CI: Confidence interval
DSM: Diagnostic and statistical manual
DIGS: Diagnostic Interview for Genetic Studies
FH: Family history
FIGS: Family Interview for Genetic Studies
GEE: General estimating equations
HR: Hazard ratio
IQR: Inter-quartile ranges
NRGR: National Institute of Mental Health Repository and Genomics Resource
OCD: Obsessive compulsive disorder
PAR: Parent
PRB: Proband
STROBE: Strengthening the Reporting of Observational Studies in Epidemiology
