# Correction to: Using density of antecedent events and trajectory path analysis to investigate family-correlated patterns of onset of bipolar I disorder: a comparison of cohorts from Europe and USA

**DOI:** 10.1186/s40345-021-00242-4

**Published:** 2021-10-26

**Authors:** Jan Scott, Florence Vorspan, Josephine Loftus, Frank Bellivier, Bruno Etain

**Affiliations:** 1 Institute of Neuroscience, Newcastle University, Newcastle upon Tyne, UK; 2 Université de Paris, Paris, France; 3 AP-HP, Département de Psychiatrie et de Médecine Addictologique, GH Saint-Louis-Lariboisière-Fernand-Widal, DMU Neurosciences Tête et Cou, Paris, France; 4 Inserm UMRS 1144, Université de Paris, Paris, France; 5 Centre Expert Trouble Bipolaire, Hospital Princesse Grace, Monaco, Monaco

## Correction to: Int J Bipolar Disord (2021) 9:29 10.1186/s40345-021-00234-4

In this article, the lines between the circles and the labels are missing in Figures 1 and 2. The correct version of Figs. 1 and 2 are given below.

**Fig. 1:**
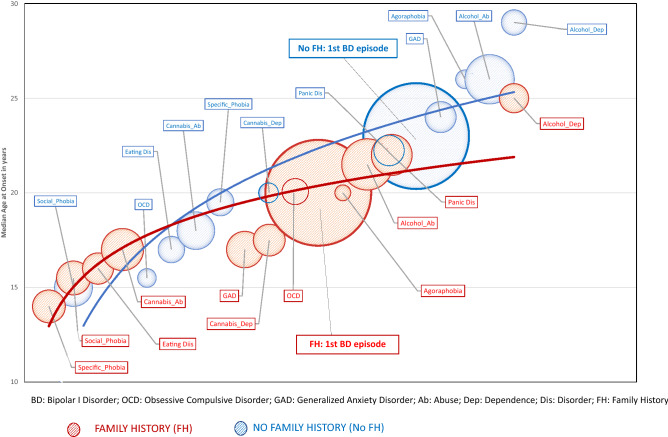
Trajectories of evolution of BD-I in groups with (FH) or without (No FH) a family history of Bipolar I Disorder (BD). In the bubble plot, the size of the bubble represents the proportion of the group who experience a particular disorder, the position of the bubble vertically gives the median AAO, whilst the position on the horizontal axis approximates to the timing of onset of a disorder in the interval between the onset of the first full threshold mental disorder and the onset of the last comorbidity. For example: in the FH subgroup, the median AAO of the first mental disorder (specific phobia) is about 13–14 years, whilst the median AAO of BD-I is about 20 years. The curve fits less well to the trajectory of comorbidities for the period post-onset of BD-I in the FH group. In the No FH group, the median AAO of the first mental disorder (social phobia) is about 14–15, whilst the median AAO of BD-I is about 22 years. In the No FH group, the curve fits less well to the trajectory path of those antecedent comorbidities with the earliest AAO. The bubbles for BD-I represent 100% in both subgroups. The location of that bubble for FH is lower on the vertical axis, indicating the disorder has an earlier AAO than in the ‘No FH’ group. Also, as can be observed, there appears to be a more obvious clustering of (relatively) early AAO antecedent comorbid disorders within a briefer time interval in the FH group, compared with the No FH group. There are a smaller number of between-group differences in the sequence of onset of different mental disorders

**Fig. 2:**
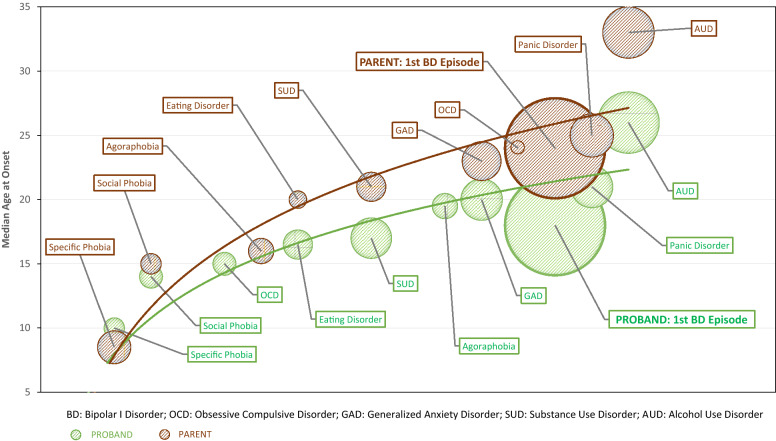
Trajectories of evolution of BD-I in Probands and Parents. In the bubble plot, the size of the bubble represents the proportion of the group who experience a particular disorder, the position of the bubble vertically gives the median AAO, whilst the position on the horizontal axis approximates to the timing of onset of a disorder in the interval between the onset of the first full threshold mental disorder and the onset of the last comorbidity. It is notable that the median AAO of the first mental disorder (specific phobia) is similar in the Proband and Parent subgroups, and that the sequence of onset of disorders (except, e.g. OCD) is similar. The size of the bubbles is somewhat similar, but AAOs of comorbidities begin to occur earlier in the Proband subgroup, and median AAO of BD-I is clearly earlier than in the Parent subgroup. Both curves show a the fit for the trajectory curve is less for comorbidities that occur post-onset of BD-I

All the changes requested are implemented in this correction and the original article (Scott et al. 2021) has been corrected.
