# Supplementary material for: Using density of antecedent events and trajectory path analysis to investigate family-correlated patterns of onset of bipolar I disorder: a comparison of cohorts from Europe and USA
Source: Int J Bipolar Disord. 2021 Oct 1;9:29. doi: 10.1186/s40345-021-00234-4 (PMC8484401; doi:10.1186/s40345-021-00234-4)
Supplement: Supplementary file 1 — Additional file 1: Appendix 1: STROBE checklists and additional details of methodology. [file 40345_2021_234_MOESM1_ESM.docx]

**Appendix 1-**

1. **STROBE checklist- European dataset**
2. **STROBE checklist- USA dataset**
3. **Additional details of study methodology**

**1. STROBE Statement- Checklist for European dataset: Cases (FH) & Controls (No FH)**

|  | Item No | Recommendation | PAGE |
| --- | --- | --- | --- |
| **Title and abstract** | 1 | (*a*) Indicate the study’s design with a commonly used term in the title or the abstract | 1 |
|  |  | (*b*) Provide in the abstract an informative and balanced summary of what was done and what was found | 2 |
| Introduction | | |  |
| Background/rationale | 2 | Explain the scientific background and rationale for the investigation being reported | 3 |
| Objectives | 3 | State specific objectives, including any prespecified hypotheses | 4 |
| Methods | | |  |
| Study design | 4 | Present key elements of study design early in the paper | 4-5 |
| Setting | 5 | Describe the setting, locations, and relevant dates, including periods of recruitment, exposure, follow-up, and data collection | 4-5 |
| Participants | 6 | (*a*) Give the eligibility criteria, and the sources and methods of selection of participants. Describe methods of follow-up | 5 & APPENDIX. Also, Loftus et al, (2020) |
|  |  | (*b*) For matched studies, give matching criteria and number of exposed and unexposed | NA |
| Variables | 7 | Clearly define all outcomes, exposures, predictors, potential confounders, and effect modifiers. Give diagnostic criteria, if applicable | 5-6 & APPENDIX |
| Data sources/ measurement | 8 | For each variable of interest, give sources of data and details of methods of assessment (measurement). Describe comparability of assessment methods if there is more than one group | 6 & APPENDIX. Also, Etain et al (2012); & Loftus et al, (2020) |
| Bias | 9 | Describe any efforts to address potential sources of bias | 6 |
| Study size | 10 | Explain how the study size was arrived at | 4-5 |
| Quantitative variables | 11 | Explain how quantitative variables were handled in the analyses. If applicable, describe which groupings were chosen and why | 6 |
| Statistical methods | 12 | (*a*) Describe all statistical methods, including those used to control for confounding | 6-7 & APPENDIX |
|  |  | (*b*) Describe any methods used to examine subgroups and interactions | 7& APPENDIX |
|  |  | (*c*) Explain how missing data were addressed | 7 & APPENDIX |
|  |  | (*d*) If applicable, explain how loss to follow-up was addressed | 7 & APPENDIX |
|  |  | (*e*) Describe any sensitivity analyses | NA |
| Results | | |  |
| Participants | 13 | (a) Report numbers of individuals at each stage of study—eg numbers potentially eligible, examined for eligibility, confirmed eligible, included in the study, completing follow-up, and analysed | 7-8 & APPENDIX |
|  |  | (b) Give reasons for non-participation at each stage | APPENDIX & AUTHORS |
|  |  | (c) Consider use of a flow diagram |  |
| Descriptive data | 14 | (a) Give characteristics of study participants (eg demographic, clinical, social) and information on exposures and potential confounders | 7-8 & APPENDIX |
|  |  | (b) Indicate number of participants with missing data for each variable of interest | APPENDIX & AUTHORS |
|  |  | (c) Summarise follow-up time (eg, average and total amount) | 7-8 & APPENDIX |
| Outcome data | 15 | Report numbers of outcome events or summary measures over time | 8 |
| Main results | 16 | (*a*) Give unadjusted estimates and, if applicable, confounder-adjusted estimates and their precision (eg, 95% confidence interval). Make clear which confounders were adjusted for and why they were included | 8-9 |
|  |  | (*b*) Report category boundaries when continuous variables were categorized | 8-9 |
|  |  | (*c*) If relevant, consider translating estimates of relative risk into absolute risk for a meaningful time period | 8-9 |
| Other analyses | 17 | Report other analyses done—eg analyses of subgroups and interactions, and sensitivity analyses | 9 & APPENDIX |
| Discussion | | |  |
| Key results | 18 | Summarise key results with reference to study objectives | 10 |
| Limitations | 19 | Discuss limitations of the study, taking into account sources of potential bias or imprecision. Discuss both direction and magnitude of any potential bias | 10-12 |
| Interpretation | 20 | Give a cautious overall interpretation of results considering objectives, limitations, multiplicity of analyses, results from similar studies, and other relevant evidence | 10-12 |
| Generalisability | 21 | Discuss the generalisability (external validity) of the study results | 10 |
| Other information | | |  |
| Funding | 22 | Give the source of funding and the role of the funders for the present study and, if applicable, for the original study on which the present article is based | 7 |

**2. STROBE Statement- Checklist for USA dataset: Cases (Probands) & Controls (Parents)**

|  | Item No | Recommendation | PAGE |
| --- | --- | --- | --- |
| **Title and abstract** | 1 | (*a*) Indicate the study’s design with a commonly used term in the title or the abstract | 1 |
|  |  | (*b*) Provide in the abstract an informative and balanced summary of what was done and what was found | 2 |
| Introduction | | |  |
| Background/rationale | 2 | Explain the scientific background and rationale for the investigation being reported | 3 |
| Objectives | 3 | State specific objectives, including any prespecified hypotheses | 4 |
| Methods | | |  |
| Study design | 4 | Present key elements of study design early in the paper | 4-5 |
| Setting | 5 | Describe the setting, locations, and relevant dates, including periods of recruitment, exposure, follow-up, and data collection | 4-5 |
| Participants | 6 | (*a*) Give the eligibility criteria, and the sources and methods of selection of participants. Describe methods of follow-up | 5 & APPENDIX |
|  |  | (*b*) For matched studies, give matching criteria and number of exposed and unexposed | APPENDIX |
| Variables | 7 | Clearly define all outcomes, exposures, predictors, potential confounders, and effect modifiers. Give diagnostic criteria, if applicable | 5-6 & APPENDIX |
| Data sources/ measurement | 8 | For each variable of interest, give sources of data and details of methods of assessment (measurement). Describe comparability of assessment methods if there is more than one group | 6 & APPENDIX; Also, NRGR & Potash et al (2007) |
| Bias | 9 | Describe any efforts to address potential sources of bias | 6 |
| Study size | 10 | Explain how the study size was arrived at | 4-5 |
| Quantitative variables | 11 | Explain how quantitative variables were handled in the analyses. If applicable, describe which groupings were chosen and why | 6 |
| Statistical methods | 12 | (*a*) Describe all statistical methods, including those used to control for confounding | 6-7 & APPENDIX |
|  |  | (*b*) Describe any methods used to examine subgroups and interactions | 7 & APPENDIX |
|  |  | (*c*) Explain how missing data were addressed | APPENDIX |
|  |  | (*d*) If applicable, explain how loss to follow-up was addressed | APPENDIX |
|  |  | (*e*) Describe any sensitivity analyses | NA |
| Results | | |  |
| Participants | 13 | (a) Report numbers of individuals at each stage of study—eg numbers potentially eligible, examined for eligibility, confirmed eligible, included in the study, completing follow-up, and analysed | 7-8 & APPENDIX |
|  |  | (b) Give reasons for non-participation at each stage | APPENDIX & AUTHORS |
|  |  | (c) Consider use of a flow diagram |  |
| Descriptive data | 14 | (a) Give characteristics of study participants (eg demographic, clinical, social) and information on exposures and potential confounders | 7-8 & APPENDIX |
|  |  | (b) Indicate number of participants with missing data for each variable of interest | APPENDIX & AUTHORS |
|  |  | (c) Summarise follow-up time (eg, average and total amount) |  |
| Outcome data | 15 | Report numbers of outcome events or summary measures over time | 8 |
| Main results | 16 | (*a*) Give unadjusted estimates and, if applicable, confounder-adjusted estimates and their precision (eg, 95% confidence interval). Make clear which confounders were adjusted for and why they were included | 8-9 |
|  |  | (*b*) Report category boundaries when continuous variables were categorized | 8-9 |
|  |  | (*c*) If relevant, consider translating estimates of relative risk into absolute risk for a meaningful time period | 8-9 |
| Other analyses | 17 | Report other analyses done—eg analyses of subgroups and interactions, and sensitivity analyses | 9 & APPENDIX |
| Discussion | | |  |
| Key results | 18 | Summarise key results with reference to study objectives | 10 |
| Limitations | 19 | Discuss limitations of the study, taking into account sources of potential bias or imprecision. Discuss both direction and magnitude of any potential bias | 10-12 |
| Interpretation | 20 | Give a cautious overall interpretation of results considering objectives, limitations, multiplicity of analyses, results from similar studies, and other relevant evidence | 10-12 |
| Generalisability | 21 | Discuss the generalisability (external validity) of the study results | 10 |
| Other information | | |  |
| Funding | 22 | Give the source of funding and the role of the funders for the present study and, if applicable, for the original study on which the present article is based | 7 |

1. **Extended Description of Methododology**

The study was approved by the French medical ethics committee (Comité de Protection des Personnes- IDRCB_AO1465_50_VI-Pitié Salpêtrière 118-08). All participants gave written informed consent and data were de-identified prior to inclusion in the study databases. Data utilized in this study was obtained from studies that followed institutional board guidance on ethical standards for research and study procedures fully complied with recommendations outlined in the revised Declaration of Helsinki.

*Selection of Databases*

A potential issue in exploring the influence of family history on the evolution of bipolar disorders (BD) and other mental disorders, is the quality of information regarding relatives of the identified proband. This was the most important issue in determining our search for appropriate datasets for the current project. We decided that using datasets used for genotyping and genetic studies, which explicitly employed recognized, valid, and reliable assessments of family history, was a priority. Furthermore, as we wished to explore the occurrence of comorbidities before and after the onset of BD, we sought datasets with a median age at interview of participants of about 45 years (as >85% cases of BD-I have an onset before this age, and most other comorbidities, expect for neurodegenerative conditions, will likely be manifest by 40-50 years). Some datasets were excluded as they did not report details of longitudinal evolution of mental health, focusing only on episodes related to mood and psychotic phenomena and not reporting on other comorbid conditions.

From the outset, we decided to focus on BD-I, as this is one of the three most reliable diagnoses in psychiatry, whilst BD-II and BD-NOS show poor reliability. So, the selection of possible datasets for the present study was further narrowed down to focus on databases that had a high proportion of BD-I cases. Lastly, although the study does not employ and combined analyses of data from the European and USA subgroups identified, we wanted to ensure that we could comment on general patterns of comorbidities. As such, the final selection was made on the basis that the identified datasets used the same or remarkably similar assessment tools and procedures for phenotyping.

*Criteria for Assembled Cohorts*

The study examines data from two assembled cohorts. Here, the latter term refers to cohorts recruited from consenting cases that are predominantly consecutive referrals to the study centres, but where we assume some preference was given to identifying individuals with a known family history. We employed a case-control design (where controls were identified as non-familial BD-I cases in one dataset and as parents of probands in the second dataset).

Eligibility criteria for inclusion of all individuals (identified as cases or controls) in the identified datasets were:

1. Best-estimate diagnosis of BD-I meeting DSM-IV criteria.
2. All comorbidities were assessed using the Diagnostic Interview for Genetic Studies (DIGS) and family history was assessed using Family Interview for Genetic Studies (FIGS).
3. The polarity of the first full-threshold episode of BD-I was recorded and the age at onset (AAO) of the first full threshold episode of major depression and of mania (or mixed state) was recorded.
4. All interviews pertaining to the longitudinal psychiatric history of the individuals were undertaken by researchers trained in the use of the clinical assessment tools and inter-rater reliability had been established and reported previously (and was known to be acceptable).
5. Basic demography (sex, date of birth) was recorded.

As noted in the next section, there were additional criteria for identifying proband-parent pairs.

We excluded individual data if any of the above details were absent and/or if there was no evidence that a full longitudinal history of comorbidities had been undertaken. We allowed individual data to be included in the study dataset if there was sporadic missing data e.g. AAO of a comorbid condition was recorded but the item stating the disorder was present or absent had not been endorsed. Likewise, if a disorder was reported as present, but the AAO was missing, we included that individual, but replaced the missing AAO with the median for that cohort.

*Identified Datasets*

Demography and phenotype information was extracted from the European genetics database (Etain et al, 2012) and a dataset from a genetic linkage study accessed via the NIMH Repository and Genomics Resource (NRGR; Bipolar Disorder distribution 12.0; access request application by Scott et al, 2018; access request confirmation reference: 5c9874082337f).

Familial and Non-Familial Cases: The European dataset was derived from the study of the ‘Genetic and Environmental Factors of Vulnerability in Bipolar Disorders’. The study database comprised information about individuals with BD who were recruited via three French university-affiliated psychiatry departments (Nancy, Bordeaux, and Paris) between 1994-2008. Individuals aged>18 were interviewed using the DIGS and FIGS. Individuals were euthymic at interview (defined as scoring <5 on the Montgomery Asberg Depression Rating Scale and <5 on the Bech-Rafaelson Mania Rating Scale; and no evidence of a major mood episodes in the last three months). Information on comorbidities and family histories was assembled from the structured clinical interviews, case notes and informants (relatives, family members and significant others). Of >700 individuals with BD, we identified 573 who had a best-estimate diagnosis of DSM IV BD-I and met all eligibility criteria (of whom 207 had a confirmed family history of BD). We noted that 24 individuals in the final sample had sporadic missing data regarding comorbidities.

Sex-Matched Proband-Parent Pairs: From datasets that included individuals with bipolar disorders we identified adults aged >18 at baseline assessment who were recruited to genetics linkage studies between 1991-2003 and were assessed using the DIGS (refs) or other structured clinical interviews (such as the Schedule for Affective Disorders and Schizophrenia-Lifetime Version). We extracted data on >400 individuals interviewed with the DIGS who had a best-estimate diagnosis of DSM-IV BD-I (mainly from the CHIP dataset; see Potash et al, 2007). From these cases, we identified probands with one or more parent who also had a diagnosis of a major mental disorder (n=297). Using the codes provided, we first excluded individuals where the parent(s) had a non-BD diagnosis. Next, we identified individuals who had a same sex biological parent who had a best-estimate diagnosis of BD-I (n=242; 126 pairs). From that group, we excluded proband-parent pairs where the second biological parent had a major psychiatric disorder (lifetime DSM IV schizophrenia, major depression, or bipolar disorder) and individuals where AAO of BD-I and/or data on longitudinal psychiatric history was not recorded in the dataset. The final cohort comprised 92 proband-parent pairs (i.e. 184 cases of BD-I). We noted that nine individuals had sporadic missing data regarding comorbidities. Also, although interviews with parents and probands were undertaken within the same study time frame, it was noteworthy that year of birth was obviously different between proband-parent pairs, so year of birth was categorized in quintiles (1940-44, !945-49, etc.) and included in multivariate analyses.

*Similarities and Differences in Data Recording*

In the European and USA datasets, the AAO of BD was defined as the age at which a patient first met DSM-IV criteria for a major depressive or manic episode (manic polarity included mixed states). The proportion of individuals with a depressive or manic onset was reported in both datasets and the AAO of the first major depressive or manic episode was recorded or could be estimated from the available information.

Both datasets reported the presence or absence of a wide range of comorbidities and both defined the AAO of comorbidities as the age at which a participant first met DSM-IV criteria for the full-threshold mental disorder.

Both datasets reported rates for specific (simple) phobia, social phobia, obsessive compulsive disorder (OCD), eating disorders, generalized anxiety disorder, any agoraphobia and panic disorder. The datasets also recorded the presence of alcohol and substance use problems. However, the European dataset included information on abuse and dependence separately, and, regarding substance use/misuse, it focused specifically on reporting comorbidity rates and AAO for cannabis abuse or dependence. The USA dataset reported categories referred to as alcohol use disorder (AUD) or substance use disorder (SUD) and reported a single AAO for each disorder (described as the reliably ascertained age for the onset of the misuse problem). Although cannabis use was a major contributor to the latter category, the comorbidity is described as SUD and the AAO is for any substance misuse problem. Data on psychotic onset of BD-I was reported in the European dataset, evidence of psychotic symptoms in mood episodes was recorded in the USA dataset, but it was not reported whether these symptoms occurred with the first BD-I major depressive or manic episode (so we could not analyze impact of this phenomenon on the USA proband-parent pairs).

Although there were some options to examine cyclothymia, dysthymia and minor depressions in the interview schedule, only some centres consistently reported data for these conditions. Given this inconsistency, it was not viable to consider incorporating existing data into the analyses or imputing data (as >70% participants in the European and USA cohort had no recordings for these variables). Importantly, the DIGS version employed by both the European and USA researchers during the decades of study lacked detailed recording of behavioural disorders or other childhood disorders or conditions that often have a childhood onset. For example, the European dataset did not report rates of conduct disorders or antisocial behaviour, etc. The USA dataset did include information on rates of ASPD, but it did not report an AAO for onset of the disorder (although it noted the age when some symptoms began to emerge). The European dataset did included information about assessment of symptoms of ADHD using the 61-item Wender Utah Rating Scale (WURS). Although this scale does have a cut-off score indicative of possible ADHD symptoms meeting full-threshold diagnostic criteria, the PPV of the WURS is modest. Also, no reliable AAO was reported for WURS symptoms or for ADHD phenomena that potentially reached the threshold for caseness.

*Constructing Illness Trajectories*

In loose terms, trajectory means “course,” and therefore illness trajectory means “course of illness” and trajectories can provide a framework for addressing patient and family expectations of what will happen regarding health and illness. For many years, psychiatry focused on trajectories post-onset of an illness, i.e., after a diagnosis was established. Such studies examined various aspects of prognosis e.g. likelihood of relapse or recurrence of illness episodes in BD, and the impact of treatments or comorbidities etc. In recent decades, research on developmental trajectories has begun to examine antecedents of a specific diagnosis as well as consequences.

The present study especially explores options for representing the path of trajectory curves, the nature of antecedents in groups with differed levels of familial risk of BD-I (which may give indications of vulnerability and resilience). As the datasets focus on reconstruction of the illness history in the assembled cohorts and all participants develop the disorder being researched, the construction of the path for the trajectory considers timing of onsets of comorbidities and BD-I as much as overall rates of onset of comorbidities and of BD-I (and of each polarity). Particularly, we wanted to include some analysis of actual the path of each trajectory (where the curve represents the trajectory passing through a given set of points) and of the variables in interest in the evolution of the disorder in an individual as defined by its position coordinates x and y (rate and AAO of each disorder), as well as a proxy representing position on the curve and momentum (i.e. speed of transition, factors that accelerate change or rate of movement ‘along the path’, etc). To achieve these goals, we first used clinical data collected using the DIGS to construct individual illness trajectories and then plotted trajectories for each subgroup, then explored accessible, easy to understand/interpret path analysis models for examining trajectory curves.

As well as classic approaches to analyses (such as survival/time dependent hazard ratios), we explored other ways of representing components of each trajectory. To do this, we estimated the path of the trajectory curve and then examined any associations with the total number of comorbid conditions that occurred per individual irrespective of AAO, and then determined the number of comorbid conditions that occurred prior to the onset of onset of BD-I per individual. Using the latter data, we estimated the *density of antecedents* of BD-I per individual (a term that refers to the number of events per year of exposure to mental disorders prior to BD-I onset, where illness exposure is calculated as the time interval between AAO of BD-I and AAO of first DSM-IV diagnosis). This variable is a proxy for intermediate stages along the trajectory curve. Each of these variables was then included in different analyses to try to capture different elements of the trajectory of onset of BD-I. It should be noted that for all estimates regarding comorbidities and densities we excluded mood episodes (e.g. if an individual reported manic onset of BD-I, we did not include major depression as a comorbidity, etc.).

*Statistical Analysis*

Analyses were undertaken separately of the European and USA datasets using RStudio version 3.5.3, supplemented by SAS version 9.4. As we regard some analyses as exploratory, we decided *a priori* that a *p* value of .05 or less would be considered statistically significant.

*Descriptive Analysis*: Basic clinical and demographic data were recorded, and characteristics of subgroup were compared using univariate analyses. Categorical data regarding rates of DSM IV disorders (which represent cumulative probabilities of meeting each diagnosis, based on DIGS best-estimate diagnoses) were described using counts and percentages and analyzed using chi-square or Fischer’s exact tests. Continuous data were described using medians with inter-quartile ranges (IQR) and analyzed using Mann Whitney U tests and McNamara and Wilcoxon signed rank tests and appropriate analyses for related samples (for the proband-parent pairs).

*Time to Event Analysis (Onset of BD-I) Analysis:* It is important to note that, as all of the individuals included in the analyses develop BD-I (i.e. by the end of the follow-up period, they all have the disorder being studied), any probability or survival curves *cannot* describe lifetime risk (as there are no unaffected individuals in the study population). Likewise, although it can be argued that rates of each comorbidity (especially as we endeavour to determine AAO) represent the cumulative incidence, we have not used this term as we are aware the data collection process (retrospective reconstruction of the evolution of mental disorders and onset of BD) means that more appropriate terminology is to call rates of disorder cumulative probability (and others might argue the best term is prevalence).

So, the initial curves represent AAO distributions for the BD I cases in each subgroup. As such, we used Kaplan Meier estimators to test for subgroup differences in the cumulative probabilities for being diagnosed with BD I as a function of age; analyses were stratified for polarity of onset and statistical significance established using Mantel-Haenszel log-rank tests.

Next, we used an accelerated failure time (AFT) model to gain insights into whether the effect of key covariates (AAO of first comorbid disorder; number of comorbidities) influenced the time to onset of BD-I. Like Cox models, AFT models are proportional hazard models, but AFT models assume that the effect of covariates may act multiplicatively with respect to the AAO, are robust to omitted covariates and are also less affected by the choice of probability distribution. As such, the estimated hazard ratios are, in effect, a time ratio with 95% confidence intervals (CI) (suggesting acceleration or deceleration in AAO of BD-I). We report HR for AAO according to polarity (which we regarded as competing risks) and consider the following covariates: subgroup, year of birth (divided into quintiles), sex, AAO of first mental disorder, and number of comorbid conditions. To explore these same phenomena in proband-parent pairs we used generalized estimating equations (GEE) to test hypotheses about AAO and the influence of covariates (GEE are recommended for correlated longitudinal data).

Eligibility criteria ensured that study participants had minimal missing data, even so, we selected analytic models that are largely robust to missing data. Additionally, we examined various options for managing the small amount of sporadic missing data, including multiple imputation etc. However, as the issue of missing data was limited, we took a conservative approach to managing this. Namely, to comply with model assumptions, if it was unclear if an individual met criteria for a specific DSM IV disorder we assumed it was absent; if AAO was unclear for an individual who met criteria for the presence of specific DSM IV disorder, we imputed the sample median AAO.

*Exploratory Analysis of Trajectories:* We used a time trend design to estimate longitudinal trends in health/illness status over time (where age represents the underlying time scale). Trend estimation related observations of interest to the time at which they occurred. Importantly, the model can be used to describe the path of the illness trajectory (without necessarily fully explaining them).

We first produced bubble plots to demonstrate the sequence and frequency of comorbidities over time (using the GraphPad Prism 8 programme). The size of each bubble represents the cumulative probability (which is the within subgroup lifetime prevalence) of a DSM IV disorder and its location on the y-axis identifies the median AAO of that disorder (the location of the bubbles on the x-axis approximates to the timing of onset of that disorder during the course of illness). Next, we introduced a logistic trendline, which is a best-fitted curve line that is particularly useful in longitudinal studies (especially when the rate of change is expected to increase or decrease quickly and then level out). The goodness of fit was estimated using the least square fitting process. This generates a value (R^2^) which represents the fraction of the variance of the explained by the fitted trendline (range is 0-1; a value of one indicates a perfect model fit). Using the algorithm described by Weaver and Wuensch, the R^2^ for subgroups were compared statistically using a Z transformation (a variation of Fisher’s r-to-z transformation; <http://cran.r-project.org/package=cocor>; this approach allows any differences between curves to be validated internally using boot strapping). To further illustrate the data represented in the plots, we undertook subgroup comparisons of number of antecedent comorbidities and density of antecedent events (separate analyses were undertaken for each cohort).

*Examples of density of antecedent calculations*:

Many studies examining the evolution of BD in various samples describe rates of comorbidities, and often examine the AAO of any full-threshold disorder that precedes the onset of BD. Prospective studies are able to report comorbidities that precede the onset of BD, as well as those that occur post-BD onset. As well as considering AAO of a first mental disorder (and its relationship to AAO of BD-I), and exploring any associations between AAO of BD-I and total number of comorbidities, we also tried to gain additional insights into individual experiences of comorbidities over time. As such, we use three measures to try to examine whether the pattern of distribution of comorbidities before and after onset of BD-I differs between subgroups and also try to capture information about an individuals’ total exposure to mental disorders (AAO of first mental disorder reported until AAO of BD-I). To illustrate this, we provide some simple examples below-

#1: an individual reports one comorbidity, but the AAO is after the AAO of BD-I; density of antecedents=0 (but number of comorbidities=1, and number of antecedents=0).

#2: an individual reports one comorbidity, the AAO is 5 years before the onset of BD-I; density of antecedents= 1/5= 0.2 (but number of comorbidities=1, and number of antecedents=1).

#3: an individual reports two comorbidities, one occurs after the onset of BD-I, the other has an AAO 10 years before the AAO of BD-I; density of antecedents=0.1 (but number of comorbidities=2, and number of antecedents=1).

#4: an individual reports three comorbidities, one occurs after the onset of BD-I, one has an AAO 5 years before the AAO of BD-I, the other has an AAO 10 years before BD-I; density of antecedents=2/10=0.2 (but number of comorbidities=3, and number of antecedents=2).

As can be seen, each variable offers a different insight into exposure to mental disorders and the relationship between comorbidities and BD-I. It is possible for individuals with different exposures to have an equivalent density, but a more complete picture can be attained by reviewing this information alongside estimates of the temporal relationships in terms of the AAO of comorbidities (before and after onset of BD-I) and the AAO of the first onset of any mental disorder in relation to the AAO of BD-I.

Also Note: We re-tested models for each cohort when data on other discrete comorbidities (e.g. on ADHD, ASPD) were incorporated. The addition of these variables (although they did not meet our specified eligibility criteria regarding DIGS recordings of presence/absence and/or AAO of full-threshold disorder) changed the magnitude of some HR, but did not alter the models significantly. The impact of incorporating data on psychosis across both cohorts was more difficult to interpret. Psychotic onset impacted somewhat on AAO of non-familial BD-I in the European dataset in the multivariate analysis (psychotic onset was associated with a non-significantly lower AAO for depressive polarity). It was not possible to reliably explore the impact of psychotic phenomena in the USA dataset as the reporting of the presence or absence of psychotic symptoms/syndromes did not include information on first AAO of these phenomena. Further, AAO of full-threshold episodes with psychotic symptoms was not reported in relation to AAO of BD-I (i.e. we were unable to ascertain whether first BD-I episode included psychotic symptoms).

**Additional References (other key references are listed in the main text)**

Diedenhofen B, Musch J. cocor: a comprehensive solution for the statistical comparison of correlations. PloS one, 2015, 10(3), e0121945.

Etain B, Lajnef M, Bellivier F, Mathieu F, Raust A, Cochet B, et al Clinical expression of bipolar disorder type I as a function of age and polarity at onset: convergent findings in samples from France and the United States. J Clin Psych, 2012, 73 (4), e561-6.

**Gill T, Gahbauer E, Han L, Allore H. Trajectories of disability in the last year of life.** N Engl J Med. **2010; 362:1173–1180.**

Harrell, Frank E. Regression modelling strategies. (Chapter 17) AFT survival regression model. London: Springer, 2015.

Ho-Trieu N, Tucker J. Another note on the use of a logarithmic time trend. Rev Mar Agri Econ. 1990, 58 (1): 89–90.

Khanal S, Sreenivas V, Acharya S. [Accelerated Failure Time Models: Application to the Survival of Acute Liver Failure Patients.](https://www.ijsr.net/archive/v3i6/MDIwMTQ0NQ==.pdf) International Journal of Science and Research 2014, 3 (6), 161 – 166.

Leckman J, Sholomskas D, Thompson W, Belanger A, Weissman M. Best estimate of lifetime psychiatric diagnosis: a methodological study. Arch Gen Psychiatry. 1982; 39:879–883.

Loftus J, Scott J, Vorspan F, Icick R, Henry C, Gard S, et al. Psychiatric comorbidities in bipolar disorders: An examination of the prevalence and chronology of onset according to sex and bipolar subtype. J Affect Disord. 2020 Apr 15; 267:258-263.

Maxwell ME. Intramural Research Program NIMH. FIGS: Family Interview for Genetic Studies. Clinical Neurogenetic Branch; 1992. Washington: NIMH.

Meeker W, Escobar L. Statistical methods for reliability data. (Chapters 4, 8, and Appendix B). Chichester: John Wiley & Sons, 2014. Pp385.

Nurnberger J, Blehar M, Kaufmann C, York-Cooler C, Simpson S, Harkavy-Friedman J, et al. Diagnostic interview for genetic studies. Rationale, unique features, and training. NIMH Genetics Initiative. Arch Gen Psych, 1994, 51, 849-859.

Parsa-Parsi R. Revised Declaration of Geneva: A Modern-Day Physician's Pledge. JAMA. 2017; 318(20):1971-1972.

Potash J, Toolan J, Steele J, Miller E, Pearl J, Zandi P, NIMH Genetics Initiative Bipolar Disorder Consortium, et al. The bipolar disorder phenome database: a resource for genetic studies. Am J Psych, 2007 Aug;164(8):1229-37.

Touloumis, A. R Package multgee: A Generalized Estimating Equations Solver for multinomial Responses. Journal of Statistical Software, 2015. 64, 1-14.

Vandenbroucke J, von Elm E, Altman D, Gotzsche P, Mulrow C, Pocock S, & STROBE initiative. Strengthening the Reporting of Observational Studies in Epidemiology (STROBE): explanation and elaboration. Ann Intern Med. 2007 Oct 16;147(8): W163-94.

Wacholder S, Mitchell G, Pee D. Selecting an efficient design for assessing exposure-disease relationships in an assembled cohort. Biometrics, 1991, 47, 1 (March), 63-76.

Weaver B, Wuensch K. SPSS and SAS programs for comparing correlations and OLS regression coefficients. Behav Res 2013, 45, 880–895

Wei, L. J. The accelerated failure time model: A useful alternative to the Cox regression model in survival analysis. Statistics in Medicine. 1992, 11 (14–15): 1871–1879.

Zeger S, Liang K. Longitudinal data analysis for discrete and continuous outcomes. Biometrics, 1986, 42, 121-130.

Zeger S, Liang K, Albert P. Models for longitudinal data: A generalized estimating equation approach. Biometrics, 1988, 44, 1049-1060.

Zheng B. Summarizing the goodness of fit on generalized models for longitudinal data. Statistics in Medicine, 2000, 19, 1265-1275.
