# Supplementary material for: Using density of antecedent events and trajectory path analysis to investigate family-correlated patterns of onset of bipolar I disorder: a comparison of cohorts from Europe and USA
Source: Int J Bipolar Disord. 2021 Oct 1;9:29. doi: 10.1186/s40345-021-00234-4 (PMC8484401; doi:10.1186/s40345-021-00234-4)
Supplement: Supplementary file 2 — Additional file 2: Appendix 2: Table S1. Comparison of rates and age at onset for each comorbidity and polarity of onset of BD-I in individuals with a family history of BD (n=209) or without a family history of BD (n=364). Table S2. Comparison of patterns of comorbidities in individuals with a family history or without a family history of BD. Table S3. Comparison of rates and age at onset for each comorbidity and polarity of onset of BD-I in probands (n=92) and their sex-matched parents (n=92). Table S4. Comparison of patterns of comorbidities in probands and parents. [file 40345_2021_234_MOESM2_ESM.docx]

Table 1S: Comparison of rates and age at onset for each comorbidity and polarity of onset of BD-I in individuals with a family

history of BD (n=209) or without a family history of BD (n=364)

|  | Cumulative Probability in % | | | Median Age at Onset in years (Interquartile Range) | | |
| --- | --- | --- | --- | --- | --- | --- |
|  | Family History of BD (n=209) | No Family History of BD (n=364) | p value^a^ | Family History of BD (n=209) | No Family History of BD (n=364) | p value^b^ |
| Specific Phobia | 9.8 | 6.7 | .202 | 14 (6.5,19.0) | 19.5 (11.5, 30.3) | .093 |
| Social Phobia | 10.3 | 12.8 | .387 | 15.5 (12.3, 19.8) | 15.0 (11.0, 20.0) | .797 |
| Obsessive Compulsive Disorder | 5.6 | 3.3 | .205 | 20.0 (18.0-27.3) | 15.5 (13.3, 19.5) | .158 |
| Generalized Anxiety Disorder | 12.0 | 8.7 | .251 | 17.0 (15.3, 25.0) | 24. 0 (18, 28.5) | .098 |
| Agoraphobia | 2.4 | 3.3 | .582 | 20.0 (14.5, 30.8) | 26.5 (17.5, 40.3) | *.046* |
| Panic Disorder | 20.1 | 18.2 | .526 | 22.0 (17, 29.8) | 22.0 (15.0, 31.0) | .985 |
| Eating Disorder | 8.8 | 6.4 | .307 | 16.0 (14.0, 21.0) | 17.0 (15.0, 23.0) | .120 |
| Cannabis Abuse | 15.9 | 12.7 | .289 | 17.0 (15.8, 19.3) | 18.0 (17.3, 21.0) | .064 |
| Cannabis Dependence | 9.2 | 3.8 | *.008* | 17.5 (16.0, 18.0) | 20.0 (18.3, 24.8) | .231 |
| Alcohol Abuse | 23.3 | 21.7 | .670 | 21.5 (18.0, 30.0) | 26.0 (18.0, 36.0) | .074 |
| Alcohol Dependence | 7.8 | 5.8 | .357 | 25.0 (18.0, 34.3) | 29.0 (23.0, 37.5) | .576 |
| Bipolar Disorder: |  |  |  |  |  |  |
| Depressive Onset | 61.3 | 57.4 | .366^d^ | 20.0 (16.0, 26.8) | 22.8 (18.0, 30.5) | *.007* |
| Manic Onset^c^ | 38.7 | 42.6 |  | 22.0 (18.0, 27.5) | 23.5 (18.0, 31.3) | .122 |
| Psychotic Symptoms at Onset^e^ | 22.5 | 24.2 | .812 | 22.5 (18.0, 28.0) | 22.0 (18.0, 31.0) | .461 |

^a^Statistical significance analysed using X^2^ or Fishers exact tests; ^b^Statistical significance analysed using Mann Whitney U test;

^c^Manic polarity includes mania and mixed states; ^d^Statistical test of proportion of depressive versus manic onsets, hence only one p value.

^e^This variable identifies individuals who reported psychotic symptoms as part of the presentation of the first episode of depression or mania.

|  | Family History of  BD =209 | No Family History of BD =364 | p value |
| --- | --- | --- | --- |
| *^a^Occurrence of Comorbidities (N, %)* |  |  | 0.01 |
| None | 89 (42.5%) | 183 (51%) |  |
| Antecedent only | 64 (31%) | 72 (20%) |  |
| Before & After BD onset | 32 (15%) | 37 (9%) |  |
| After BD onset only | 24 (11.5%) | 72 (20%) |  |
| *Grouped Median (IQR)* |  |  |  |
| ^b^Number of Comorbidities per individual | 1.3 (0, 2) | 0.8 (0, 2) | 0.10 |
| ^b,c^Number of Antecedents per individual | 1.1 (0, 2) | 0.47 (0, 1) | 0.04 |
| ^b,c^Density of Antecedents per individual | .078 (.027, .124) | .051 (.019, .103) | 0.018 |
| ^d^Density: Mean (SD) | 0.072 (0.022) | 0.055 (0.020) |  |
| ^a^Analyzed using chi-squared; ^b^Analyzed using MWU or Wilcoxon;  ^c^Data were log transformed; ^d^For information | | | |

Table 2S: Comparison of patterns of comorbidities in individuals with a family history or without a family history of BD

Table 2S: Comparison of rates and age at onset for each comorbidity and polarity of onset of BD-I in probands (n=92)

and their sex-matched parents (n=92)

|  | Cumulative Probability in % | | | Median Age at Onset in years (Interquartile Range) | | |
| --- | --- | --- | --- | --- | --- | --- |
|  | Probands (n=92) | Parents (n=92) | p value^a^ | Probands (n=92) | Parents (n=92) | p value^b^ |
| Specific Phobia | 4.3 | 10.9 | .095 | 10.0 (7.5, 15.0) | 8.5 (5.5, 10.3) | .328 |
| Social Phobia | 5.4 | 4.3 | .733 | 14.0 (8.0, 20.0) | 15.0 (10.0, 19.8) | .861 |
| Obsessive Compulsive Disorder | 5.4 | 1.1 | .067 | 15.0 (10.8, 27.8) | 24.0* | ns |
| Generalized Anxiety Disorder | 17.4 | 15.0 | .838 | 20.0 (16.3, 27.0) | 23.3 (17.5, 30.3) | .247 |
| Agoraphobia | 6.5 | 6.5 | 1.00 | 19.5 (12.3, 25.8) | 16.6 (11.8, 30.3) | .510 |
| Panic Disorder | 17.4 | 18.5 | .874 | 21.0 (16.0, 25.3) | 25.0 (14.8, 40.5) | .264 |
| Eating Disorder | 8.6 | 3.2 | .174 | 16.5 (12.0, 20.8) | 20.0 (13.8, 26.3) | .078 |
| Substance Use Disorder | 16.3 | 8.6 | .090 | 17.0 (15.3, 22.8) | 21.3 (18.0, 28.3) | .128 |
| Alcohol Use Disorder | 37 | 26 | .076 | 26.0 (21.3, 34.8) | 33.0 (25.3, 40.5) | .052 |
| Bipolar Disorder: |  |  |  |  |  |  |
| Depressive Onset | 61 | 61 | 1.00^d^ | 17.3 (13.0, 22.8) | 23.0 (17.0, 34.0) | *.001* |
| Manic Onset^c^ | 39 | 39 |  | 20.0 (16.8, 27) | 24.3 (17.8, 35.8) | *.001* |

Statistical analyse are shown to allow some comparison to the European dataset. However, it should be born in mind that these univariate tests do not formally control for correlated data; the multivariate analyses of the proband-parent pairs are therefore the more reliable analyses.

^a^Statistical significance analysed using McNamara tests; ^b^Statistical significance analysed using Wilcoxon tests;

^c^Manic polarity includes mania and mixed states; ^d^Statistical test of proportion of depressive versus manic onsets, hence only one p value.

*Only one case was reported in the parent group.

Table 4S: Comparison of patterns of comorbidities in probands and parents

|  | **Proband = 92** | **Parent = 92** | p value |
| --- | --- | --- | --- |
| *^a^Occurrence of Comorbidities (N, %)* |  |  | 0.1 |
| None | 41 (45%) | 48 (52%) |  |
| Antecedent only | 31 (34%) | 30 (33%) |  |
| Before & After BD onset | 16 (17%) | 11 (12%) |  |
| After BD onset only | 4 (4%) | 3 (4%) |  |
| *Grouped Median (IQR)* |  |  |  |
| ^b^Number of Comorbidities per individual | 1.33 (0,2) | 1.04 (0,2) | 0.09 |
| ^b,c^Number of Antecedents per individual | 1.21 (0,2) | .96 (0,2) | 0.13 |
| ^b,c^Density of Antecedents per individual | .088 (.052, .115) | .071 (.032, .126) | 0.04 |
| ^d^Density: Mean (SD) | 0.076 (0.022) | 0.06 (0.020) |  |
| ^a^Analyzed using chi-squared; ^b^Analyzed using McNamara or Wilcoxon tests;  ^c^Data were log transformed; ^d^Provided for information | | | |
