# Supplementary material for: Using density of antecedent events and trajectory path analysis to investigate family-correlated patterns of onset of bipolar I disorder: a comparison of cohorts from Europe and USA
Source: Int J Bipolar Disord. 2021 Oct 1;9:29. doi: 10.1186/s40345-021-00234-4 (PMC8484401; doi:10.1186/s40345-021-00234-4)
Supplement: Supplementary file 3 — Additional file 3: Appendix 3: Figure S1. Trajectory paths for FH and non-FH. Figure S2. Trajectory plots for Probands and Parents. [file 40345_2021_234_MOESM3_ESM.pdf]

FIGURE 2S (A): Trajectory paths for FH and non-FH

In a bubble plot, the size of the bubble represents the proportion of the group who experience a particular disorder, the position of the bubble vertically gives the median AAO, whilst the position on the horizontal axis approximates to the timing of onset of a disorder in the interval between the onset of the first mental disorder identified and the onset of the last mental disorder.

a) FH of BD: The equations for the trajectory paths is: y=7.35 ln(x) + 0.599

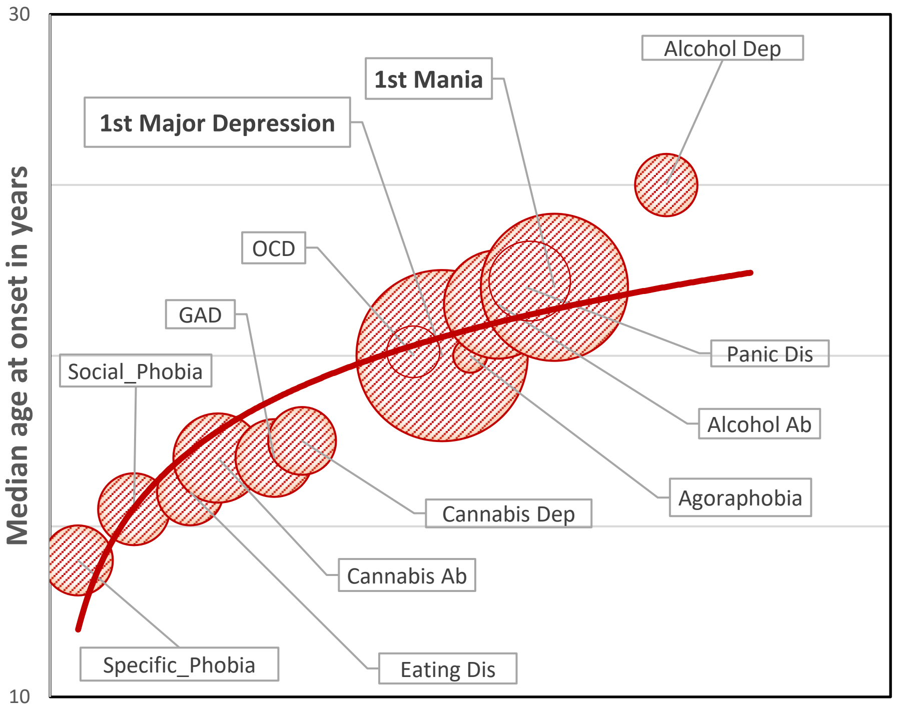

b) No FH of BD: The equations for the trajectory paths is: y= 8.21 ln(x) + 1.033

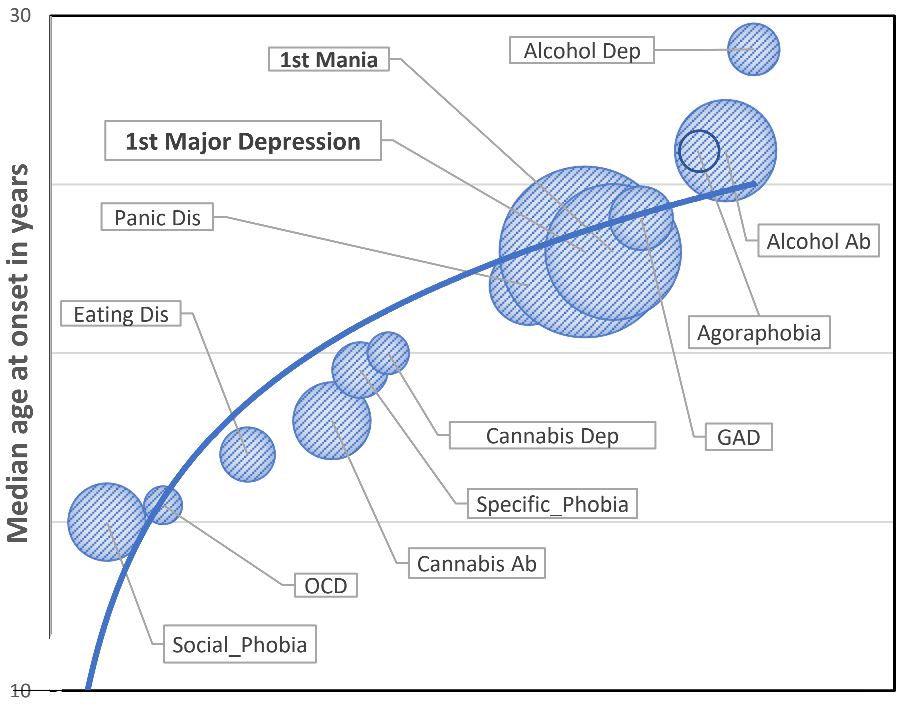

OCD: Obsessive Compulsive Disorder; GAD: Generalized Anxiety Disorder; Dep: Dependence; Ab: Abuse; Dis: Disorder

FIGURE 2S (B): Trajectory plots for Probands and Parents

In a bubble plot, the size of the bubble represents the proportion of the group who experience a particular disorder, the position of the bubble vertically gives the median AAO, whilst the position on the horizontal axis approximates to the timing of onset of a disorder in the interval between the onset of the first mental disorder identified and the onset of the last mental disorder.

a) Probands : The equations for the trajectory paths is: y=6.98 ln(x) + 3.03

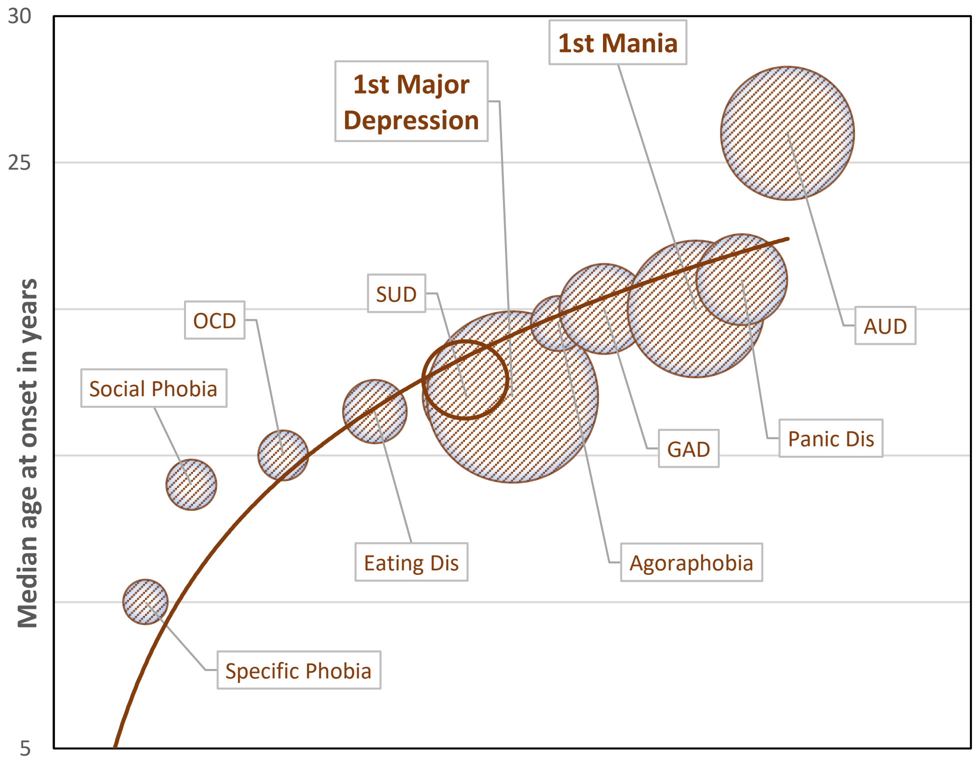

b) Parents : The equations for the trajectory paths is: y= 9.286 ln(x) + 1.43

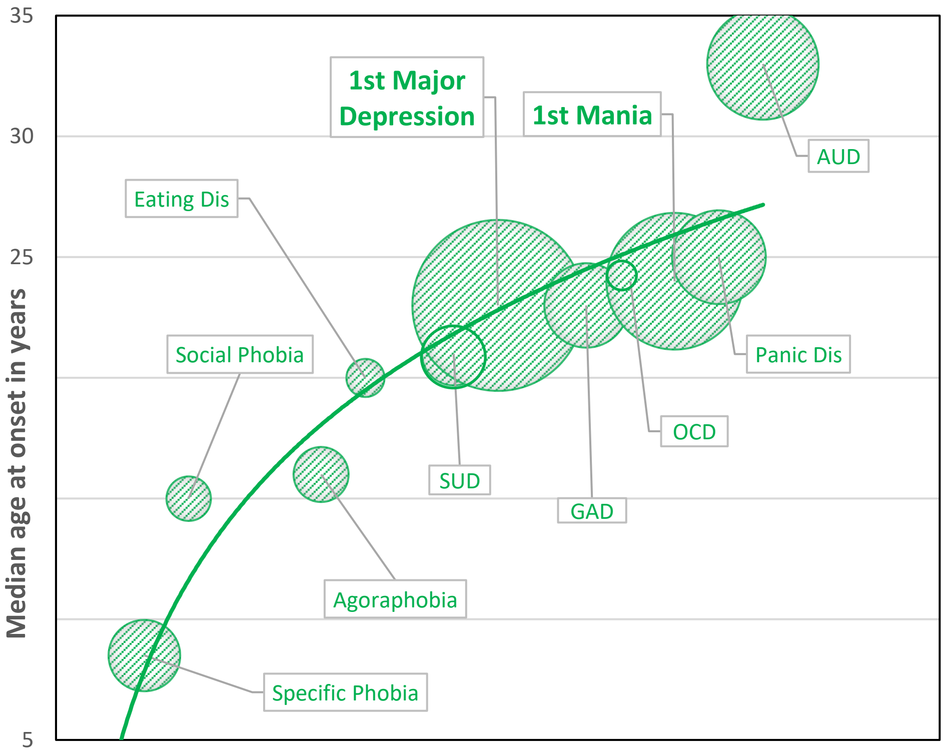

OCD: Obsessive Compulsive Disorder; SUD: Substance Use Disorder; AUD: Alcohol Abuse Disorder; GAD: Generalized Anxiety Disorder; Dis: Disorder
